# Rheology of Indian Honey: Effect of Temperature and Gamma Radiation

**DOI:** 10.1155/2014/935129

**Published:** 2014-10-14

**Authors:** Sudhanshu Saxena, Lata Panicker, Satyendra Gautam

**Affiliations:** ^1^Food Science and Safety Section, Food Technology Division, Bhabha Atomic Research Centre, Mumbai 400085, India; ^2^Solid State Physics Division, Bhabha Atomic Research Centre, Mumbai 400085, India

## Abstract

Honey brands commonly available in Indian market were characterized for their rheological and thermal properties. Viscosity of all the honey samples belonging to different commercial brands was found to decrease with increase in temperature (5–40°C) and their sensitivity towards temperature varied significantly as explained by calculating activation energy based on Arrhenius model and ranged from 54.0 to 89.0 kJ/mol. However, shear rate was not found to alter the viscosity of honey indicating their Newtonian character and the shear stress varied linearly with shear rate for all honey samples. Honey is known to contain pathogenic microbial spores and in our earlier study gamma radiation was found to be effective in achieving microbial decontamination of honey. The effect of gamma radiation (5–15 kGy) on rheological properties of honey was assessed, and it was found to remain unchanged upon radiation treatment. The glass transition temperatures (*T*_*g*_) of these honey analyzed by differential scanning calorimetry varied from −44.1 to −54.1°C and remained unchanged upon gamma radiation treatment. The results provide information about some key physical properties of commercial Indian honey. Radiation treatment which is useful for ensuring microbial safety of honey does not alter these properties.

## 1. Introduction

Rheological property is one of the most important physical attributes that could affect texture, sensory rating as well as other quality parameters including shelf stability during storage [1, 2]. Color, flavor, texture, and nutritive value are other parameters determining the acceptability criteria of any food [3]. Honey is a natural viscous food well known for its high nutritional and prophylactic values [4]. Viscosity is an important quality attribute of honey and there are various physical as well as biochemical factors such as temperature, moisture content, and presence of crystals, colloids, and sugars that influence this property. From rheological point of view, honey is a material with changing molecular structure and honey viscosity affects the interactions with and within the microenvironment, material transport, and also the consumption experience [5, 6]. Viscosity affects sensorial properties of honeys and so the acceptability of consumers [1]. This property is of great practical importance to beekeepers and honey processors as the knowledge of honey rheology is necessary in the area of process engineering that involves different stages including handling, storage, processing, quality control, and transportation. The flow behavior properties as indicated by rheological profiling provides an indirect measure of product consistency and quality, which is the guiding factor for the design of mechanics of honey processing [7]. The effect of temperature on rheological properties also needs to be documented because a wide variation in temperature is encountered during processing and storage of liquid foods [8]. The viscosity of honey generally decreases with the increase in the temperature. Considerable research work has been carried out to understand the exact nature of the viscosity variation with temperature. Several models have been proposed to describe the temperature-viscosity relationship of foods including Arrhenius model and Williams-Landel-Ferry (WLF) model [9–11]. The knowledge of glass transition temperature (*T*_*g*_) is essential in assuring quality, stability, and safety of various food products [12].

Most of the honeys were reported to have Newtonian fluid like characteristics, whereas, some honey has been reported as non-Newtonian fluids [13]. Some fluids also exhibit a change in viscosity with time at a constant shear rate and classified as thixotropic, where the fluid viscosity decreases with time and rheopectic where the fluid viscosity increases with time.

Radiation processing, a cold process, is used as a method for preservation of food commodities and is finding increasing applications [14, 15]. In our earlier study complete microbial decontamination of honey was achieved by gamma radiation treatment at 10 kGy for most of the honey samples, however, in a few samples, higher dose of 15 kGy was required [16]. The current study deals with evaluation of the rheological and thermal properties of commercial Indian honey and analyzing the effect of gamma radiation treatment on these properties. The availability of data for such study is very limited worldwide, and in Indian context it is not available.

## 2. Materials and Methods

### 2.1. Honey Samples and Radiation Treatment

The present study was carried out using seven commercial Indian honey brands. Fresh honey weighing 250 g, packed and sealed in glass bottles, was purchased from a local market and stored at 4°C. The storage period did not exceed more than 6 months for any of the honey brands. The honey samples were kept at ambient temperature (26 ± 2°C) overnight before analysis was performed. Gamma radiation treatment was carried out in a cobalt-60 Gamma Chamber-5000 (GC-5000, source strength Co^60^: 260 Tbq, BRIT, Mumbai, India, dose rate 6.5 kGy/h) at Bhabha Atomic Research Centre, Mumbai, India. Standard chemical ceric-cerous sulfate dosimeters were used to measure absorbed radiation dose employing electrochemical cell method [17]. The dose uniformity ratio was 1.03. A 50 mL aliquot of honey was packed and sealed in high-density polyethylene packets and treated with different doses of gamma radiation (5–15 kGy) at ambient temperature. Nonirradiated honey served as control.

### 2.2. Rheological Analysis

The rheological properties of the honey were evaluated using a Physica MCR 301 rheometer from Anton Paar (Anton Paar GmbH, Austria, Europe) which has a parallel plate geometry set-up with a fixed lower plate and an upper plate (referred to as geometry) that can rotate or oscillate. Measurements were carried out at eight different temperatures between 5 and 40°C and at shear rate ranging from 1.49 to 149 s^−1^. As the viscosity of honey can be affected by the presence of air bubbles, therefore, the samples were preconditioned before the rheological analysis. The samples were incubated at 50°C for 1 h in a water bath and then kept for 15 h at 30°C in an incubator. Then the samples were placed in the measuring element of the rheometer and thermostated to reach the desired temperature of measurement. The rheological data was analysed using the RheoPlus software.

The effect of temperature on the apparent viscosity of honey was analyzed using the Arrhenius equation:

(1) $$\begin{array}{c} \eta =A_{0}\;e^{\left(E_{a}/RT\right)}, \end{array}$$

where *η* is the viscosity (Pa·s), *A*_0_ is a material constant (Pa·s), *E*_*a*_ is the flow activation energy (J/mol), *R* is the gas constant (*R* = 8.31 J/mol K), and *T* is the absolute temperature (K). The material constant (preexponential factor in Arrhenius equation) represents viscosity at a temperature approaching infinity [18]. The Arrhenius parameters were determined through linear regression by plotting the inverse of temperature (1/*T*) on *x*-axis and the corresponding observed log viscosity values (ln⁡⁡*A*) on *y*-axis. Straight line equation (*y* = *mx* + *c*) corresponding to (1) in logarithmic form, ln⁡⁡*A* = ln⁡⁡*A*_0_ + *E*_*a*_/*R*(1/*T*), was obtained, and the *A*_0_ and *E*_*a*_ values for all the samples were calculated.

The mean absolute percentage error (MA%E), which indicates the deviance of the observed values from the calculated viscosity values using the Arrhenius equation, was calculated using the following formula:

(2) $$\begin{array}{c} \left[\text{MA}\%\text{E}=\left(\frac{100}{n}\right)\Sigma \left(\frac{Y_{0}-Y_{C}}{Y_{C}}\right)\right], \end{array}$$

where *Y*_0_ is the observed value, *Y*_*C*_ is the calculated value, and *n* represents the number of pairs of samples [10, 19].

### 2.3. Glass Transition Analysis

The glass transition in honey was determined using a differential scanning calorimeter (Mettler Toledo AG, 822c, Switzerland) with empty aluminum pan as reference. In principle, differential scanning calorimetry (DSC) measures the difference between the heat flows from the sample and reference sides of a sensor as a function of temperature or time. Differences in heat flow arise when a sample absorbs or releases heat due to thermal effects. The calorimeter was attached with a cooling system (liquid N_2_) which efficiently controlled and monitored temperature up to −150°C. Temperature and enthalpy calibration of the instrument were done using cyclohexane and indium. Approximately 31–35 mg honey samples were hermetically sealed in aluminium sample pans and analysed by calorimetry under continuous flow of dry N_2_ gas (60 mL/min) to avoid condensation of moisture. Before recording the heating scan the sample was first heated to 50°C then cooled to −130°C. Then these samples were scanned between temperature range −130°C to 50°C at the scanning rate of 10°C/min to obtain the complete thermal behavior of honey. The presence of the glass transition in the cooling run and the repeated heating cycle indicate the reversibility of the glass transition. The STAR^e^ software (Mettler Toledo) was used to obtain the glass transition temperature (*T*_*g*_).

### 2.4. Statistical Analysis

The experiments were repeated in three independent sets, each in triplicate, and observations were analyzed and expressed in terms of mean and standard deviation (SD) by taking all data points in consideration. The mean values were compared using one-way ANOVA (analysis of variance) at the level of significance, where *P* ≤ 0.05. The correlation value was determined using the Correl function of Microsoft Excel.

## 3. Results and Discussion

### 3.1. Rheological Characteristics of Indian Honey

Rheology is used as a quality parameter for many food products and is also an important property for honey [20]. The effect of temperature and radiation processing on honey were assessed and the findings are discussed herewith.

#### 3.1.1. Dependence of Honey Viscosity on Temperature

**Figure 1:**
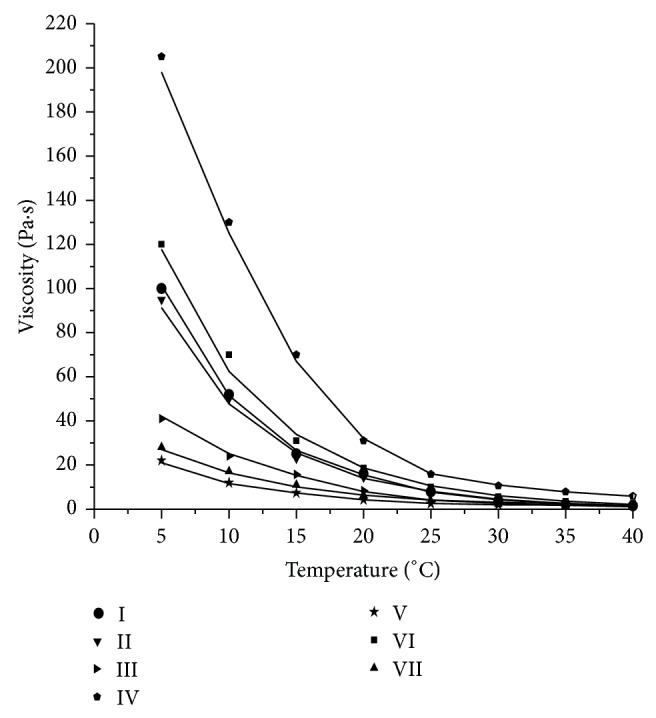
Effect of temperature on viscosity of different commercial Indian honey (I–VII).

The viscosities of all the seven honey were measured at temperatures ranging from 5°C to 40°C and found to decrease with increase in the temperature (Figure 1). Temperature dependent decrease in viscosity of honey may be attributed to reduced molecular friction and hydrodynamic forces. All honey followed the same pattern of change in the viscosity with increase in temperature and satisfactorily followed the Arrhenius relationship. In all cases the determination coefficient (*R* ^2^) exceeded values >0.99. The mean absolute percentage error varied from 3.7 to 8.4 which is below 10%, that is, the upper limit on the acceptability of MA%E as mentioned by Kleijnen [21]. In a recent study on seven varieties of unifloral Australian honey, Bhandari et al. [10] found similar observation. The effect of temperature was found to be more pronounced up to 30°C. However, at temperatures above 30°C, the differences in viscosity are very small in most of the honey analysed. At higher temperatures the difference in viscosity among seven honey brands decreases but still exists even at 40°C which could be attributed to the natural variations in composition (sugar, colloid materials, and water content) [22]. The values of flow activation energy of the analyzed honey ranged from 54.2 to 88.8 kJ/mol and the decreasing order of the activation energy for the brands is I > II > *VI*⁡>IV > III > VII > V (Table 1). Activation energy (*E*_*a*_) reflects the sensitivity of viscosity to temperature changes; higher *E*_*a*_ means that the viscosity is relatively more sensitive to a temperature change [23]. Therefore, the viscosity of the brand I is relatively highly sensitive, whereas, the viscosity of brand VII is least sensitive to the temperature changes. In a recent study on honey produced from different fruit plants of northern India *E*_*a*_ was found to vary from 63.63 to 81.48 kJ/mol [24]. The *E*_*a*_ of Greek and Polish honeys varied between 69.1–93.8 kJ/mol and 92.3–105.3 kJ/mol, respectively [18, 25]. The *E*_*a*_ of the Turkish honey ranged from 63.4 to 78.5 kJ/mol [26]. An inverse correlation value of 0.65 was found to exist between *E*_*a*_ and moisture content. A similar negative correlation value (*r* = −0.61) was also reported between *E*_*a*_ and moisture content for Greek honey by Lazaridou et al. [25].

#### 3.1.2. Dependence of Honey Viscosity on Shear Rate

**Figure 2:**
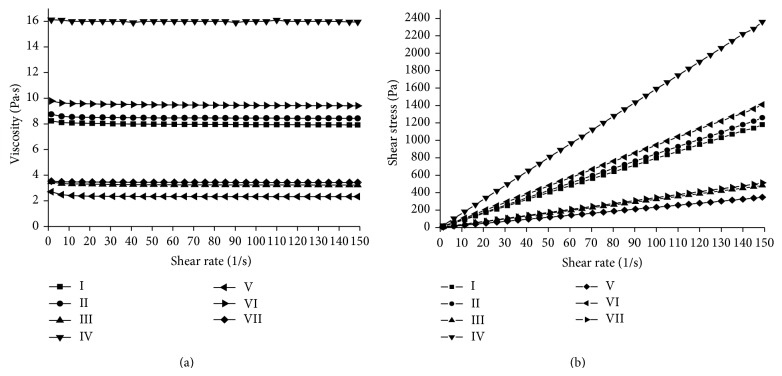
(a) Viscosity of honey as a function of shear rate at the temperature of 25°C. (b) Shear stress for honey at variable shear rates at 25°C.

Figure 2(a) illustrates the viscosity values as a function of shear rate for honey at 25°C. The apparent viscosity remained constant with increasing shear rate. Besides, the shear stress was always found to be a linear function of shear rate (Figure 2(b)). These observations indicated Newtonian flow behavior of honey, which has also been reported in case of Indian honey by other authors too [24, 27]. The Newtonian behavior has also been shown for the Chinese natural honey between the temperature ranges of 10–25°C [28]. None of the analyzed Indian honey showed non-Newtonian behavior although there are reports of the non-Newtonian behavior in some Indian honey which may due to the presence of colloidal particles [29, 30]. The non-Newtonian behavior is not observed in simple liquids of low molecular weight, but is observed in complex fluids like colloids, emulsions, macromolecular solutions, and gels where there are discrete entities at the micro/nanoscale [31].

#### 3.1.3. Effect of Gamma Radiation on Honey Viscosity

When a physical treatment such as gamma radiation is applied to food some changes on its viscosity may occur but contrary to the belief, in a dose range of 5–15 kGy, gamma radiation was not found to affect the viscosity of the honey studied (Table 2). Similar observations were also reported for the viscosity of Brazilian honey irradiated up to 10 kGy [32]. Gamma radiation treatment being a cold physical process does not result in any significant increase in the temperature of the sample being irradiated. Just after irradiation (15 kGy) of honey, a marginal increase from 28°C to 35°C was observed, which came down to 28°C after 40 min of storage at ambient temperature [16]. The composition of honey is considered to be responsible for its rheological property [33]. Carbohydrates are the major constituents of honey and about 90% of these are monosaccharides followed by small amount of other disaccharides and trisaccharides [34]. Although gamma radiation can cause chain scission and breakage of glycosidic bond resulting in general degradation of the polymers and affect the viscosity, but in case of honey due to the lack of significant level of higher poly- or oligosaccharides and/or other polymeric substances, possibilities of radiation induced depolymerization leading to altered viscosity are very less [14, 15].

### 3.2. Glass Transition Temperature

The glass transition temperature of honey varied from −44.1 to −54.1°C, where moisture content varied from 17.2–21.6 g/100 g (Table 3). The thermal behavior of honey is shown in Supplementary Figure 3 (Supplementary data available online at http://dx.doi.org/10.1155/2014/935129). The highest *T*_*g*_ (−44.1°C) was observed for brand II, whereas, lowest *T*_*g*_ was noticed for brand V (−54.1°C). The *T*_*g*_ values obtained in the current study are in good agreement with the literature values [35]. As expected an inverse correlation value of ~0.50 was observed for *T*_*g*_ with moisture content and viscosity. The glass transition temperature is a function of both moisture content and the type of solute [36]. It is known that the glass transition temperature shifts to lower temperatures with increase in the moisture content as plasticization caused by water results in depression of the *T*_*g*_ of completely amorphous and partially crystalline food products due to the ability of water molecules to weaken hydrogen bonds, dipole-dipole, and intra- and intermolecular interactions [37].

Gamma radiation treatment was not found to affect the glass transition temperature of honey (Table 3), which could be due to the insignificant change in physicochemical composition of honey upon gamma radiation treatment as reported in our earlier study [16]. In general, low molecular weight fragments produced by the scission reactions decrease the glass transition temperature of the polymer matrix [38]. However, it is felt that due to the lack of significant level of higher poly- or oligosaccharides and/or other polymeric substances in honey, radiation mediated change in *T*_*g*_ values is very insignificant. To the best of our knowledge this is the first report on the effect of gamma radiation on the glass transition of honey.

## 4. Conclusion

All the commercial Indian honey brands evaluated showed Newtonian behavior and the viscosity of honey remained unaltered upon gamma radiation treatment. The study indicated that Arrhenius equation can be suitably used to predict the viscosity values of the honey in the current study. Interestingly gamma radiation (15 kGy) was also not found to affect the glass transition temperature of honey.

## Supplementary Material

The figure 3 shows the thermal scans of honey (I-VII) indicating glass transition as obtained by differential scanning calorimeter.

## Figures and Tables

**Table 1:** Parameters for Arrhenius model for Indian honey.

Honey brand	*A*_0_ (Pa·s)	*E*_*a*_ (kJ/mol)	*R* ^2^	MA%E^a^
I	2.0 × 10^−15^	89.0	0.9955	6.9
II	1.1 × 10^−14^	85.0	0.9939	8.4
III	6.0 × 10^−12^	68.0	0.9983	4.2
IV	4.5 × 10^−13^	78.0	0.9973	3.7
V	1.2 × 10^−9^	54.0	0.9963	5.3
VI	2.9 × 10^−14^	83.0	0.9966	7.7
VII	1.4 × 10^−11^	66.0	0.9962	5.4

^a^Mean absolute percentage error.

**Table 2:** Effect of gamma radiation treatment on viscosity (Pa·s ± SD^x^) of honey measured at 25°C.

Honey brand	Control	5 kGy	7.5 kGy	10 kGy	15 kGy
I	7.9^b^ ± 1.0	8.0^b^ ± 0.8	8.0^b^ ± 1.0	7.9^b^ ± 1.2	7.9^b^ ± 1.0
II	8.5^b^ ± 0.4	8.3^b^ ± 0.03	8.3^b^ ± 0.3	8.4^b^ ± 0.2	8.4^b^ ± 0.4
III	3.6^c^ ± 0.3	3.5^c^ ± 0.2	3.5^c^ ± 0.2	3.5^c^ ± 0.1	3.5^c^ ± 0.4
IV	16.0^a^ ± 1.0	16.0^a^ ± 0.6	15.8^a^ ± 1.0	15.8^a^ ± 0.4	16.0^a^ ± 1.0
V	2.4^d^ ± 0.3	2.3^d^ ± 0.1	2.3^d^ ± 0.3	2.3^d^ ± 0.2	2.3^d^ ± 0.2
VI	9.6^b^ ± 0.3	9.6^b^ ± 0.1	9.6^b^ ± 1.0	9.6^b^ ± 0.7	9.6^b^ ± 0.2
VII	3.4^c^ ± 0.1	3.4^c^ ± 1.0	3.3^c^ ± 1.0	3.3^c^ ± 0.7	3.3^c^ ± 1.0

^x^Standard deviation; ^a–d^letters depict the significance of differences in mean values when analyzed by one-way analysis of variance (ANOVA). Same letter across the row or column indicates insignificant differences, whereas different letters indicate significant differences (*P* ≤ 0.05).

**Table 3:** Glass transition temperature (*T*_*g*_) of different honey and effect of gamma radiation treatment.

Honey brand	*T*_*g*_ (°C) of nonirradiated honey	*T*_*g*_ (°C) of irradiated (15 kGy) honey
I	−47.9 ± 0.54	−47.9 ± 0.54
II	−44.1 ± 0.44	−44.1 ± 0.54
III	−47.1 ± 0.36	−47.1 ± 0.36
IV	−48.0 ± 0.48	−47.4 ± 0.48
V	−54.1 ± 0.56	−53.9 ± 0.56
VI	−44.2 ± 0.73	−43.2 ± 0.68
VII	−51.0 ± 0.54	−51.4 ± 0.54
