# Supplementary figures and images for: Rheology of Indian Honey: Effect of Temperature and Gamma Radiation
Source: Int J Food Sci. 2014 Oct 14;2014:935129. doi: 10.1155/2014/935129 (PMC4745488; doi:10.1155/2014/935129)

## Supplementary data

Fig. 3 DSC thermal scans of honey showing glass transition

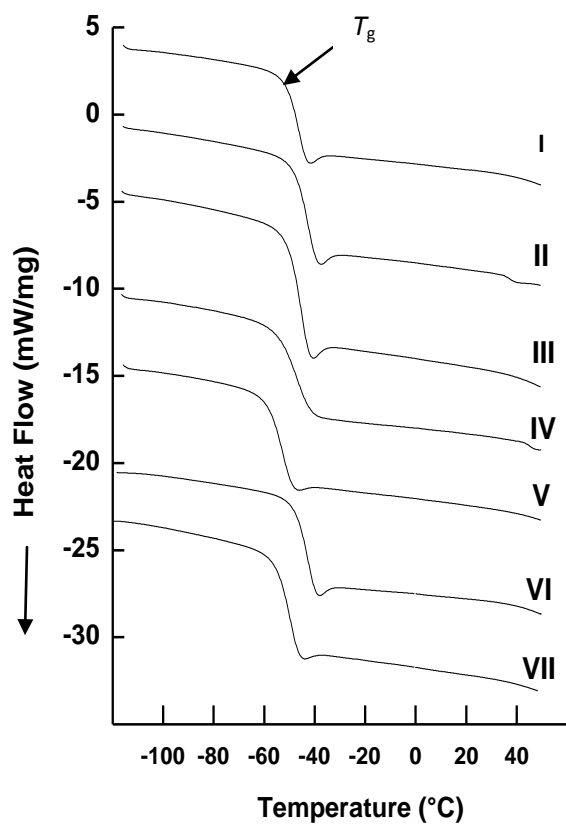

Supplement: Supplementary file 1 — The figure 3 shows the thermal scans of honey (I-VII) indicating glass transition as obtained by differential scanning calorimeter. [file 935129.f1.pdf]
